# Supplementary material for: Effects of Physical Exercises and Verbal Stimulation on the Functional Efficiency and Use of Free Time in an Older Population under Institutional Care: A Randomized Controlled Trial
Source: J Clin Med. 2020 Feb 9;9(2):477. doi: 10.3390/jcm9020477 (PMC7074187; doi:10.3390/jcm9020477)
Supplement: Supplementary file 1 [file jcm-09-00477-s001.zip › Supplemenmtary files/Supplementary files.docx]

**Table S1.** Clinical characteristics of the participants.

|  |  | **BE (n=51)** | **BE+VS (n=51)** | **FET (n=51)** | **FET+VS (n=51)** | **p-value** |
| --- | --- | --- | --- | --- | --- | --- |
|  |  |  | Number (%)  Mean (SD) | |  |  |
| **Other Outcomes** |  |  |  |  |  |  |
| **Postural Balance Assessment**  **(eyes open)** | SP [mm] | 514.16 (503.81) | 519.80 (303.21) | 557.18 (518.54) | 539.20 (273.55) | p=0.952 |
|  | SPAP [mm] | 425.04 (464.09) | 399.31 (243.44) | 456.96 (472.87) | 434.65 (248.73) | p=0.891 |
|  | SPML [mm] | 245.76 (401.44) | 247.57 (175.04) | 231 (185.91) | 253.80 (163.51) | p=0.973 |
|  | MA [mm] | 5.58 (3.09) | 6.08 (3.61) | 5.87 (3.19) | 7.72 (8.36) | p=0.141 |
|  | MAAP [mm] | 4.26 (2.75) | 4.25 (2.25) | 4.37 (2.64) | 5.78 (7.53) | p=0.219 |
|  | MAML [mm] | 2.74 (1.57) | 3.34 (3.21) | 2.86 (2.25) | 3.85 (3.81) | p=0.184 |
|  | MaxAP [mm] | 16.81 (9.54) | 18.49 (11.04) | 18.73 (14.34) | 23.09 (22.89) | p=0.201 |
|  | MaxML [mm] | 12.82 (12.42) | 18.14 (21.39) | 12.64 (10.49) | 21.36 (26.43) | p=0.053 |
| **Postural Balance Assessment**  **(eyes closed)** | SP [mm] | 556.98 (660.25) | 566.14 (410.20) | 609.22 (721.61) | 569.04 (482.06) | p=0.971 |
|  | SPAP [mm] | 470.51 (578.83) | 464.10 (357.80) | 515.56 (638.92) | 477.84 (417.62) | p=0.958 |
|  | SPML [mm] | 211.88 (228.74) | 235.47 (185.41) | 229.2 (250) | 213.16 (199.80) | p=0.931 |
|  | MA [mm] | 5 (3.28) | 4.92 (2.85) | 5.00 (3.36) | 5.01 (3.05) | p=0.999 |
|  | MAAP [mm] | 4.06 (2.91) | 4.03 (2.61) | 4.05 (2.91) | 3.99 (2.14) | p=0.999 |
|  | MAML [mm] | 2.1 (1.46) | 2.08 (1.39) | 2.09 (1.50) | 2.36 (2.08) | p=0.790 |
|  | MaxAP [mm] | 17.2 (12.3) | 16.15 (10.91) | 18.39 (13.31) | 17.02 (13.09) | p=0.842 |
|  | MaxML [mm] | 9.56 (11.27) | 7.49 (4.85) | 8.78 (8.68) | 8.51 (7.36) | p=0.658 |

SP, total path length; SPAP, statokinesiogram path length; SPML, statokinesiogram path length; MA, mean COP displacement; MAAP, mean COP displacement; MAML, mean COP displacement; MaxAP, maximal COP displacement; MaxM. maximal COP displacement.

**Table S2.** Mean difference scores for each group across time.

|  |  | **BE** | **BE + VS** | **FET** | **FET+ VS** | **BE** | **BE + VS** | **FE** | **FE + VS** |
| --- | --- | --- | --- | --- | --- | --- | --- | --- | --- |
|  |  | Baseline – 12 weeks | | | | Baseline – 24 weeks | | | |
| **Other Outcomes** |  |  |  |  |  |  |  |  |  |
| **Postural Balance Assessment**  **(eyes open)** | SP [mm] | -37.63 | -80.39 | -78.16 | -76.31 | -58.00 | -35.45 | -18.02 | -41.76 |
|  |  | (-206.08; 130.83) | (-120.87; -39.91) * | (-184.20; 27.88) | (-128.51; -24.12) * | (-182.90; 66.90) | (-81.69; 10.79) | (-117.05; 81.01) | (-118.54; 35.01) |
|  | SPAP [mm] | -39.39 | -96.86 | -100.84 | -106.67 | -90.82 | -60.08 | -52.73 | -111.84 |
|  |  | (-204.86; 126.08) | (-138.45; -55.28) * | (-207.53; 5.84) | (-154.84; -58.50) * | (-200.91; 19.26) | (-115.54; -4.62) * | (-143.40; 37.95) | (-175.16; -48.52) * |
|  | SPML [mm] | -35.53 | 8.39 | 9.06 | -3.75 | 63.92 | 92.33 | 115.27 | 47.22 |
|  |  | (-127.06; 56.00) | (-18.33; 35.12) | (-16.18; 34.30) | (-41.23; 33.74) | (-49.48; 177.32) | (52.02; 132.65) * | (64.72; 165.82) * | (-15.18; 109.62) |
|  | MA [mm] | 0.15 | -1.16 | -0.63 | -1.93 | 0.18 | -0.45 | -0.02 | -2.01 |
|  |  | (-1.49; 1.79) | (-2.09; -0.23) * | (-1.53; 0.27) | (-3.89; 0.04) | (-1.49; 1.85) | (-1.11; 0.22) | (-0.80; 0.75) | (-4.10; 0.09) |
|  | MAAP [mm] | -0.34 | -1.10 | -0.79 | -1.70 | -0.46 | -0.70 | -0.50 | -2.35 |
|  |  | (-1.90; 1.21) | (-1.76; -0.45) * | (-1.43; -0.16) * | (-3.53; 0.12) | (-1.47; 0.54) | (-1.28; -0.11) * | (-1.14; 0.14) | (-4.35; -0.35) * |
|  | MAML [mm] | 0.45 | -0.26 | 0.40 | -0.54 | 0.88 | 0.49 | 0.64 | -0.27 |
|  |  | (-0.22; 1.12) | (-0.99; 0.46) | (-0.42; 1.22) | (-1.42; 0.34) | (-0.38; 2.14) | (-0.03; 1.01) | (-0.12; 1.40) | (-1.13; 0.60) |
|  | MaxAP [mm] | -0.77 | -3.15 | -4.36 | -6.48 | -1.35 | -2.48 | -2.23 | -6.56 |
|  |  | (-7.56; 6.01) | (-6.56; 0.25) | (-8.13; -0.59) * | (-11.69; -1.27) * | (-5.74; 3.05) | (-5.02; 0.05) | (-6.44; 1.98) | (-12.15; -0.98) * |
|  | MaxML [mm] | -0.10 | -2.42 | 0.50 | -3.60 | 2.54 | -0.35 | 5.12 | -3.67 |
|  |  | (-3.36; 3.16) | (-8.13; 3.29) | (-2.24; 3.24) | (-9.44; 2.24) | (-2.91; 7.99) | (-5.62; 4.91) | (-2.15; 12.39) | (-8.90; 1.55) |
| **Postural Balance Assessment**  **(eyes closed)** | SP [mm] | -72.43 | -93.37 | -60.43 | -58.93 | -92.24 | -92.84 | -40.24 | -122.67 |
|  |  | (-243.06; 98.20) | (-170.18; -16.57) * | (-203.25; 82.39) | (-169.02; 51.16) | (-233.06; 48.59) | (-169.16; -16.52) * | (-195.05; 114.58) | (-227.28; -18.05) * |
|  | SPAP [mm] | -124.49 | -106.06 | -63.80 | -83.49 | -122.84 | -132.16 | -85.00 | -179.04 |
|  |  | (-253.75; 4.77) | (-190.21; -21.91) * | (-159.58; 31.97) | (-174.18; 7.20) | (-249.21; 3.52) | (-212.49; -51.82) * | (-218.78; 48.78) | (-281.02; -77.06) * |
|  | SPML [mm] | 7.29 | 50.53 | 20.71 | 29.96 | 83.08 | 78.73 | 83.29 | 26.27 |
|  |  | (-46.61; 61.2) | (7.64; 93.42) * | (-25.77; 67.18) | (-25.17; 85.09) | (25.72; 140.43) * | (46.42; 111.03) * | (28.14; 138.45) * | (-21.53; 74.08) |
|  | MA [mm] | -0.86 | -0.72 | -0.08 | -0.35 | -0.33 | -0.22 | 0.04 | -1.05 |
|  |  | (-1.67; -0.04) * | (-1.33; -0.11) * | (-0.74; 0.59) | (-1.23; 0.52) | (-1.23; 0.56) | (-0.93; 0.49) | (-1.24; 1.32) | (-1.72; -0.38) * |
|  | MAAP [mm] | -0.81 | -0.96 | -0.16 | -0.63 | -0.48 | -0.73 | 0.01 | -1.18 |
|  |  | (-1.57; -0.05) * | (-1.46; -0.47) * | (-0.74; 0.42) | (-1.23; -0.03) * | (-1.21; 0.26) | (-1.30; -0.17) * | (-0.94; 0.96) | (-1.82; -0.55) * |
|  | MAML [mm] | 0.39 | 0.28 | 0.52 | -0.02 | 0.65 | 0.61 | 0.96 | -0.05 |
|  |  | (-0.08; 0.86) | (-0.19; 0.76) | (0.07; 0.96) * | (-0.64; 0.61) | (0.13; 1.17) * | (0.09; 1.14) * | (0.06; 1.87) * | (-0.66; 0.56) |
|  | MaxAP [mm] | -7.19 | -4.92 | -6.80 | -7.43 | -6.03 | -4.38 | -3.24 | -6.72 |
|  |  | (-10.60; -3.79) * | (-7.92; -1.92) * | (-10.6; -3.00) * | (-10.68; -4.18) * | (-9.45; -2.61) * | (-7.78; -0.97) * | (-10.30; 3.82) | (-10.13; -3.31) * |
|  | MaxML [mm] | 1.04 | 1.18 | 0.49 | 0.27 | -1.12 | 3.46 | 2.19 | 0.41 |
|  |  | (-4.93; 7.00) | (-1.02; 3.38) | (-2.76; 3.73) | (-2.95; 3.49) | (-4.67; 2.43) | (-0.27; 7.18) | (-2.04; 6.42) | (-3.08; 3.90) |

* statistically significant result. SP, total path length; SPAP, statokinesiogram path length; SPML, statokinesiogram path length; MA, mean COP displacement; MAAP, mean COP displacement; MAML, mean COP displacement; MaxAP, maximal COP displacement; MaxML, maximal COP displacement.

**Table S3.** Between-group comparisons in postural balance at 12 weeks.

|  |  | **Post hoc (Bonferroni) Analysis** | | | | | | **ANOVA  p value** |
| --- | --- | --- | --- | --- | --- | --- | --- | --- |
|  |  | BE  vs  BE + VS | BE  vs  FET | BE  vs  FET + VS | BE + VS vs  FET | BE + VS  vs  FET + VS | FET  vs  FE T+ VS |  |
| **Other Outcomes** |  |  |  |  |  |  |  |  |
| **Postural Balance Assessment**  **(eyes open)** | SP [mm] | p=1.000 | p=1.000 | p=1.000 | p=1.000 | p=1.000 | p=1.000 | p=0.928 |
|  | SPAP [mm] | p=1.000 | p=1.000 | p=1.000 | p=1.000 | p=1.000 | p=1.000 | p=0.775 |
|  | SPML [mm] | p=1.000 | p=1.000 | p=1.000 | p=1.000 | p=1.000 | p=1.000 | p=0.595 |
|  | MA [mm] | p=0.981 | p=1.000 | p=0.246 | p=1.000 | p=1.000 | p=0.981 | p=0.215 |
|  | MAAP [mm] | p=1.000 | p=1.000 | p=0.803 | p=1.000 | p=1.000 | p=1.000 | p=0.495 |
|  | MAML [mm] | p=0.778 | p=1.000 | p=0.439 | p=0.778 | p=1.000 | p=0.445 | p=0.191 |
|  | MaxAP [mm] | p=1.000 | p=1.000 | p=0.628 | p=1.000 | p=1.000 | p=1.000 | p=0.429 |
|  | MaxML [mm] | p=1.000 | p=1.000 | p=1.000 | p=1.000 | p=1.000 | p=1.000 | p=0.547 |
| **Postural Balance Assessment**  **(eyes closed)** | SP [mm] | p=1.000 | p=1.000 | p=1.000 | p=1.000 | p=1.000 | p=1.000 | p=0.98 |
|  | SPAP [mm] | p=1.000 | p=1.000 | p=1.000 | p=1.000 | p=1.000 | p=1.000 | p=0.844 |
|  | SPML [mm] | p=1.000 | p=1.000 | p=1.000 | p=1.000 | p=1.000 | p=1.000 | p=0.658 |
|  | MA [mm] | p=1.000 | p=0.848 | p=1.000 | p=1.000 | p=1.000 | p=1.000 | p=0.444 |
|  | MAAP [mm] | p=1.000 | p=0.674 | p=1.000 | p=0.387 | p=1.000 | p=1.000 | p=0.276 |
|  | MAML [mm] | p=1.000 | p=1.000 | p=1.000 | p=1.000 | p=1.000 | p=0.842 | p=0.496 |
|  | MaxAP [mm] | p=1.000 | p=1.000 | p=1.000 | p=1.000 | p=1.000 | p=1.000 | p=0.711 |
|  | MaxML [mm] | p=1.000 | p=1.000 | p=1.000 | p=1.000 | p=1.000 | p=1.000 | p=0.985 |

SP, total path length; SPAP, statokinesiogram path length; SPML, statokinesiogram path length; MA, mean COP displacement; MAAP, mean COP displacement; MAML, mean COP displacement; MaxAP, maximal COP displacement; MaxML, maximal COP displacement.

|  |  | **Post hoc (Bonferroni) Analysis** | | | | | | **ANOVA p value** | |
| --- | --- | --- | --- | --- | --- | --- | --- | --- | --- |
|  |  | BE vs  BE + VS | BE vs  FET | BE vs  FET + VS | BE + VS vs  FET | BE + VS vs  FET + VS | FET vs  FET + VS | |  |
| **Other Outcomes** |  |  |  |  |  |  |  |  | |
| **Postural Balance Assessment**  **(eyes open)** | SP [mm] | p=1.000 | p=1.000 | p=1.000 | p=1.000 | p=1.000 | p=1.000 | p=0.941 | |
|  | SPAP [mm] | p=1.000 | p=1.000 | p=1.000 | p=1.000 | p=1.000 | p=1.000 | p=0.721 | |
|  | SPML [mm] | p=1.000 | p=1.000 | p=1.000 | p=1.000 | p=1.000 | p=1.000 | p=0.552 | |
|  | MA [mm] | p=1.000 | p=1.000 | p=0.189 | p=1.000 | p=0.493 | p=0.254 | p=0.127 | |
|  | MAAP [mm] | p=1.000 | p=1.000 | p=0.160 | p=1.000 | p=0.207 | p=0.160 | p=0.080 | |
|  | MAML [mm] | p=1.000 | p=1.000 | p=0.419 | p=1.000 | p=0.924 | p=0.750 | p=0.296 | |
|  | MaxAP [mm] | p=1.000 | p=1.000 | p=0.527 | p=1.000 | p=0.779 | p=0.779 | p=0.323 | |
|  | MaxML [mm] | p=1.000 | p=1.000 | p=0.671 | p=0.745 | p=1.000 | p=0.207 | p=0.172 | |
| **Postural Balance Assessment**  **(eyes closed)** | SP [mm] | p=1.000 | p=1.000 | p=1.000 | p=1.000 | p=1.000 | p=1.000 | p=0.816 | |
|  | SPAP [mm] | p=1.000 | p=1.000 | p=1.000 | p=1.000 | p=1.000 | p=1.000 | p=0.700 | |
|  | SPML [mm] | p=1.000 | p=1.000 | p=0.606 | p=1.000 | p=0.606 | p=0.606 | p=0.279 | |
|  | MA [mm] | p=1.000 | p=1.000 | p=1.000 | p=1.000 | p=1.000 | p=0.559 | p=0.375 | |
|  | MAAP [mm] | p=1.000 | p=1.000 | p=0.769 | p=0.769 | p=1.000 | p=0.135 | p=0.140 | |
|  | MAML [mm] | p=1.000 | p=1.000 | p=0.675 | p=1.000 | p=0.675 | p=0.183 | p=0.173 | |
|  | MaxAP [mm] | p=1.000 | p=1.000 | p=1.000 | p=1.000 | p=1.000 | p=1.000 | p=0.701 | |
|  | MaxML [mm] | p=0.512 | p=1.000 | p=1.000 | p=1.000 | p=1.000 | p=1.000 | p=0.330 | |

**Table S4.** Between-group comparisons in postural balance at 24 weeks.

SP, total path length; SPAP, statokinesiogram path length; SPML, statokinesiogram path length; MA, mean COP displacement; MAAP, mean COP displacement; MAML, mean COP displacement; MaxAP, maximal COP displacement; MaxML, maximal COP displacement.
